# Performance of the EUROIMMUN Anti-SARS-CoV-2 ELISA Assay for detection of IgA and IgG antibodies in South Africa

**DOI:** 10.1371/journal.pone.0252317

**Published:** 2021-06-23

**Authors:** Maemu P. Gededzha, Nakampe Mampeule, Sarika Jugwanth, Nontobeko Zwane, Anura David, Wendy A. Burgers, Jonathan M. Blackburn, Jurette S. Grove, Jaya A. George, Ian Sanne, Lesley Scott, Wendy Stevens, Elizabeth S. Mayne

**Affiliations:** 1 Department of Immunology, Faculty of Health Sciences, University of the Witwatersrand, Johannesburg, South Africa; 2 National Health Laboratory Services, Johannesburg, South Africa; 3 Department of Molecular Medicine and Haematology, School of Pathology, Faculty of Health Sciences, University of Witwatersrand, Johannesburg, South Africa; 4 Division of Medical Virology, Department of Pathology, Faculty of Health Sciences, University of Cape Town, Cape Town, South Africa; 5 Wellcome Centre for Infectious Diseases Research in Africa, Cape Town, South Africa; 6 Institute of Infectious Disease and Molecular Medicine, University of Cape Town, Cape Town, South Africa; 7 Divisions of Chemical and System Biology, Department of Integrative Biomedical Sciences, Faculty of Health Sciences, University of Cape Town, Cape Town, South Africa; 8 Department of Chemical Pathology, Faculty of Health Sciences, University of the Witwatersrand, Johannesburg, South Africa; 9 Clinical HIV Research Unit, Department of Medicine, Faculty of Health Sciences, University of the Witwatersrand, Johannesburg, South Africa; Consiglio Nazionale delle Ricerche, ITALY

## Abstract

Severe Acute Respiratory Syndrome-Coronavirus 2 (SARS-CoV-2) has been identified as the causative agent for causing the clinical syndrome of COVID -19. Accurate detection of SARS-CoV-2 infection is not only important for management of infected individuals but also to break the chain of transmission. South Africa is the current epicenter of SARS-CoV-2 infection in Africa. To optimize the diagnostic algorithm for SARS-CoV-2 in the South African setting, the study aims to evaluate the diagnostic performance of the EUROIMMUN Anti-SARS-CoV-2 assays. This study reported the performance of EUROIMMUN enzyme-linked immunosorbent assay (ELISA) for semi-quantitative detection of IgA and IgG antibodies in serum and plasma samples targeting the recombinant S1 domain of the SARS-CoV-2 spike protein as antigen. Samples were collected from 391 individuals who had tested positive for SARS-CoV-2 and 139 SARS CoV-2 negative controls. Samples were stratified by number of days’ post-PCR diagnosis and symptoms. The sensitivity of EUROIMMUN IgG was 64.1% (95% CI: 59.1–69.0%) and 74.3% (95% CI: 69.6–78.6%) for IgA and the specificity was lower for IgA [84.2% (95% CI: 77–89.2%)] than IgG [95.2% (95% CI: 90.8–98.4%)]. The EUROIMMUN Anti-SARS-CoV-2 ELISA Assay sensitivity was higher for IgA but low for IgG and improved for both assays in symptomatic individuals and at later timepoints post PCR diagnosis.

## Introduction

In December 2019, pneumonia of unknown etiology was reported in a cluster of patients linked to a sea food market in Wuhan City, Hubei Province of China [1]. The causal agent was later identified as a new strain of coronavirus named Severe Acute Respiratory Syndrome-Coronavirus 2 (SARS-CoV-2), causing the clinical syndrome of COVID -19. The World Health Organization declared COVID-19 a pandemic on March 11, 2020. As of 08 February2021, over 100 million cases of SARS-CoV-2, with over 1 million deaths have been reported globally. South Africa is the current epicenter of aSARS-CoV-2 infection in Africa with over 1 million cases and more than 40, 000 deaths [2].

The incubation period of SARS-CoV-2 ranges from 2 to 14 days [3, 4]. The majority of infected patients show mild symptoms, with approximately 10–20% of cases progressing to severe or critical disease [5]. Major risk factors for severe disease include older age and co-morbidities such as hypertension, diabetes, chronic obstructive pulmonary disease (COPD), and cardiovascular disease [6, 7].

SARS-CoV-2 is a single-stranded RNA virus belonging to the family Coronaviridae and the genus *Betacoronavirus* [1]. Its genome consists of approximately 29, 000 nucleotides (nt) with 14 open reading frames (ORFs) encoding 27 proteins, flanked by 5’ and 3’ untranslated region segments. The genome contains four structural proteins [spike surface glycoprotein (S), small envelope protein (E), matrix protein (M), and nucleocapsid protein (N)], eight accessory proteins and 15 non-structural (ns) proteins [8]. The major antigenic targets are the S and N proteins and the antibody response is primarily directed towards these antigens although antibodies can be detected against all 4 structural proteins [9]. The S protein (S1) contains the receptor-binding domain (RBD) which binds the receptor on the host cell, the ACE-2 receptor, for viral entry [10], when the virus initially infects epithelial cells in the nasopharynx [10, 11]. The N protein comprises the ribonucleoprotein core and is important for packaging [12].

Although the reverse transcription-polymerase chain reaction (RT-PCR) is the gold standard for diagnosis of acute SARS-CoV-2 infection [13], there are a number of limitations of these assays including the inability to detect past infection. The sensitivity of the PCR assay also declines at about 14 days post-symptom onset and some studies have raised concerns about potential contamination with subsequent false positive testing [14–17]. Serology testing can detect past infection and increases in sensitivity at later timepoints post-infection especially for the S protein while the, antibodies against the N protein wane overtime while the S protein persist over time [18]. It may also assist in determining the immune status of individuals [19, 20]. Serological tests for COVID-19 detect specific antibodies against SARS-CoV-2 antigens. IgM is produced in response to the initial exposure to an antigen, followed by isotype switching to IgG. IgA, may be produced earlier and by more individuals than either IgM or IgG [21]. The first point of entry of SARS-CoV-2 is the mucosal surface and IgA plays an important role as part of mucosal immunity [22]. Studies have found IgA to be possess the capacity of viral neutralization and may be important for protection against SARS-CoV-2 and for vaccine efficacy [23, 24].

The immunological response that mediates protective immunity to SARS-CoV-2 is not well-understood. High antibody titers correspond to severity of disease and do not necessarily indicate protection from reinfection or sterility [25, 26]. To date, multiple formal and rapid serological assays for SARS CoV-2 have been developed which detect IgA, IgM and IgG antibodies. These tests have shown uneven performance in studies globally [27] and the clinical utility of serological testing as a diagnostic tool is incompletely understood. There are limited data on the use of these assays in African populations where antibody responses may be affected by previous vaccinations and a high burden of both communicable and non-communicable diseases. Finally, the kinetics of the antibody response suggest that the optimal use of many formal assays is at timepoints at least 14 days after symptom onset [28].

The EUROIMMUN Anti-SARS-CoV-2 IgA and IgG assay test kits (EUROIMMUN Medizinische Labor diagnostika AG, Lübeck, Germany) [29, 30] are enzyme-linked immunosorbent assays (ELISA) that provide semi-quantitative serology results against the S1 domain of the spike protein of SARS-CoV-2 in serum or plasma (EDTA, lithium heparin or sodium citrate plasma). The RBD within S1 domain has been identified as the target of neutralizing antibodies with a possibility of protective immunity and neutralizing antibodies elicited upon vaccination [31–36]. A correlation between anti-S antibodies and plasma neutralization has been demonstrated in several studies [37–40]. The EUROIMMUN Anti-SARS-CoV-2 IgA and IgG assays can be automated allowing relatively high throughput of testing. Both the IgA and the IgG assays have been validated in a number of studies across the world and have shown variable performance (S1 and S2 Tables).

The Immunology Laboratory at the National Health Laboratory Service, Braamfontein is a tertiary referral laboratory with expertise in serology testing. To optimize the diagnostic algorithm for SARS-CoV-2 in the South African setting, this laboratory undertook to validate high throughput serology assays for the African population including the EUROIMMUN Anti-SARS-CoV-2 IgA and IgG assays.

## Methods

### Study population

The study population consist of voluntary participants >18 years who were recruited to participate in the research “Fourway validation of serological and rapid point-of-care testing for Severe Acute Respiratory Viral-2 Coronavirus (SARS-CoV2 or COVID-19) in South Africa”. Information about the research and its purpose were explained to participants and they were asked to sign the written informed consent form. Only participants who have signed the written informed consent form were included in the study. Participants were requested to complete a questionnaire regarding their, demographic information symptomatology and to declare any comorbidities, medications, and travel history (S1 Appendix). No information by which samples can be traced back to patients were released or published. Participants who tested positive (n = 391) by RT-qPCR were included in the study. Informed consent was obtained to collect up to 2 tubes of EDTA venous blood and 2–6 tubes of venous blood in serum separator tubes. This study was approved by the human research ethics committee of the University of Witwatersrand (M200468).

Participants were requested to complete a questionnaire regarding their symptomatology and to declare any comorbidities.

Samples were transported at ambient temperature within 4 hours of collection to the immunology laboratory at the National Health Laboratory Service, Braamfontein campus. All samples were assigned a study specific identifier number. Serum tubes were centrifuged at 3500rpm for 15 minutes and plasma tubes were centrifuged at 800rpm on either a ROTINA 420R or 460R centrifuges (Andreas Hettich GmbH & Co. KG, Tuttlingen, Germany). Serum and plasma were collected and stored at -80°C in 200 μl aliquots.

SARS CoV-2 negative controls (n = 139) were defined as:

1. Individuals who tested negative for RT-PCR on at least 2 occasions and tested negative by Western Blot and multiplexed immunofluorescence serology for all viral proteins
2. Stored serum samples from banks which were constituted prior to February 2020
3. Stored serum samples from patients with confirmed autoimmune diseases with high polyspecific antibody production including rheumatoid arthritis and systemic lupus erythematosus
4. Serum samples from patients with pneumonia of viral aetiology diagnosed prior to 2020.

Fresh peripheral blood samples were processed in the same way as positive samples. Serum or plasma was extracted and assigned a study identifier number. Frozen serum samples were thawed once only and run immediately.

### EUROIMMUN IgG and IgA testing

The assay kit is an ELISA test utilizing a plate with wells coated with recombinant S protein antigen. The samples testing was performed automatically by the Euroimmun analyser as per manufacture’s instructions. Briefly, samples were first diluted 1:101 in sample buffer provided with the kit. 100μl each of the calibrator, positive and negative controls or diluted patient samples was then transferred into the individual microplate wells and incubated for 60 minutes at +37 °C ± 1 °C. Reagent wells were washed 3 times with 450μl of working-strength wash buffer. 100μl of enzyme conjugate (peroxidase-labelled anti-human IgA) was added into each of the microplate wells and incubated for 30 minutes at +37°C ± 1°C. Reagent wells were washed again 3 times with 450μl of working-strength wash buffer. 100μl of chromogen/substrate solution was added into each of the microplate wells and incubated for 30 minutes at room temperature (+18°C to +25°C). Finally, 100μl of stop solution was added into each of the microplate wells and photometric measurement of the colour intensity was made at a wavelength of 450 nm and a reference wavelength between 620 nm and 650 nm within 30 minutes of adding the stop solution.

Results are reported semi-quantitatively by calculation of a ratio of the extinction of the control or patient sample over the extinction of the calibrator. This ratio is interpreted as follows:

- <0.8– negative
- ≥ 0.8to<1.0 –borderline
- ≥ 1– positive.

### Confirmatory tests

Additional serological tests were performed on a subset of the SARS-CoV-2 samples (n = 199) using in-house ELISAs, immunofluorescence assays and Western blots, as described below.

#### Enzyme-linked immunosorbent assay (ELISA)

ELISA assays were carried out to detect IgG binding to the S1 domain of the spike protein, as previously described [41]. This ELISA method was adapted from an FDA-approved protocol [42], with the modification that S1 was cloned, expressed and purified from *Nicotiana benthamiana* plants. Following coating of 96-well plates (Nunc MaxiSorp, Thermo Fisher, Waltham, Massachusetts, United States) with purified recombinant S1, individual patient samples were diluted 1:50 in PBS and added for 2 h at room temperature; detection was via a goat anti-human IgG (Fc-specific) peroxidase conjugate, as described [41] Thresholds for true positive signal were determined using the mean plus 2 standard deviations of the data from pre-pandemic controls (n = 58).

#### In-house immunofluorescence assays

For the in-house immunofluorescence and western blot assays, SARS-CoV-2 nucleocapsid (N) and spike (S) proteins were cloned and expressed in insect cells as fusions to a biotin carboxyl carrier protein (BCCP) tag. Briefly, the structural domains of the N protein (core domains, residues 44–362; N-terminal domain, residues 43–179; C-terminal domain, residues 246–363; Uniprot ID P0DTC9) and the full-length S ectodomain protein (residues 1–1273; including a stabilising R682S mutation; Uniprot ID P0DTC2) were synthesized (GeneArt, Thermo Fisher, Waltham, Massachusetts, United States) and cloned in to a proprietary *Escherichia coli/ Spodoptera frugiperda* transfer vector, pPRO8, such that the constructs encoded the N protein domains or S ectodomain as in-frame fusions to a C-terminal Biotin Carboxyl Carrier Protein (BCCP) and c-Myc tag. pPRO8 is a derivative of pTriEx1.1 (Novagen) (Sigma-Aldrich, Temecula, California, United States) and encodes the *E*. *coli* BCCP domain (amino acids 74–156 of the *E*. *coli accB* gene product) downstream of a viral polyhedrin promoter and cloning sites; baculoviral 603 gene and the 1629 genes flank this *polh*-BCCP expression cassette to enable homologous recombination of the construct into a replication-deficient baculoviral genome [43]. Following co-transfection of *S*. *frugiperda* Sf9 cells with the relevant pPRO8-derived transfer vector and a replication deficient bacmid vector (*Autographa californica* baculovirus vector pBAC10:KO_1629_), baculovirus was amplified to P1 stocks and BCCP-tagged recombinant proteins were expressed in *S*. *frugiperda* superSf9-3 strain (Oxford Expression Technologies) for 3 days at 27°C (Thermo Fisher, Waltham, Massachusetts, United States), according to previously published protocols [43]. During expression, the BCCP tag becomes *in vivo* biotinylated by the host biotin ligase only if the fusion partner folds correctly [43].

After 3 days, insect cells were harvested and resuspended in lysis buffer (25 mM Hepes, 50 mM KCL, 20% glycerol, 0.1% Triton X100, 1X Halt^™^ Protease Inhibitor Cocktail, EDTA-free (Thermo Fisher, Waltham, Massachusetts, United States), 0.25% sodium deoxycholate acid, 25 U/mL Pierce Universal nuclease (Thermo Fisher, Waltham, Massachusetts, United States), pH 8). Expression yields and *in vivo* biotinylation of the N domains and S ectodomain were assessed by western blot using a streptavidin-HRP conjugate probe (GE Healthcare, Chicago, Illinois, United states of America). Lysates were stored at -80°C until use. Crude insect cell lysates for the N protein N-terminal and C-terminal domains, along with relevant controls (BCCP tag only; biotinylated human IgG, IgA, and IgM (Rockland, Limerick, PA); & insect cell lysate only) were printed in triplicate on proprietary streptavidin-coated hydrogel slides (7.5 x 2.5 cm) by Sengenics Corporation, using piezo-electric printing technology (Biodot, Irvine, California, United States of America). with a mean spot size of 125 μm. Twenty-four replica arrays were printed per slide. After printing, the slides were blocked (20% Glycerol, 25 mM HEPES buffer (pH 7.4), 50 mM KCl, 1% Triton X-100, 1 mM DTT and 50 μM Biotin) and stored at 4°C until use. Through this process, the biotinylated, BCCP-tagged N protein domains became immobilised and purified *in situ* in a single step, with all non-biotinylated proteins washing away [44]. The resultant multi-epitope SARS-CoV-2 nucleocapsid protein microarray platform has been demonstrated to provide 100% sensitivity and 100% specificity is distinguishing severe COVID-19 cases from controls [45]. Individual arrays were isolated using ProPlate 24-plex multi-well chambers (GraceBio, Bend, Oregon, United States of America), after which patient samples (n = 199) were diluted 1:50 in assay buffer (PBST, 0.1% BSA, 0.1% milk powder), added to individual chambers on replica arrays and incubated for 1 hour at room temperature. The chambers were then rinsed with 1 × PBST, after which the slides were removed from the gaskets and washed 3 × 5 mins in 1 × PBST, then dried by centrifugation at 1200 × g for 2 mins. Slides were then incubated with fluorescently-labelled detection antibody (20 μg/ml Cy3-labelled anti-human IgG in PBST, 0.1% BSA, 0.1% Milk powder) for 30 mins at RT, washed 3 × 5 mins in 1 × PBST, dried by centrifugation at 1200 × g and scanned at a fixed gain setting on an InnoScan 710 microarray scanner (10μm resolution) (Innopsys, Carbonne, France). The median foreground and local background pixel intensities for each spot were extracted and quantified automatically (Mapix v9) (Innopsys, Carbonne, France), after which the mean net fluorescence intensity of each spot was calculated as the difference between the raw mean intensity and its local background. Thresholds for true positive signal were determined using the mean plus 2 standard deviations of the data from pre-pandemic controls (n = 88) assayed on replica microarrays under the same conditions, which gave a calculated specificity in this cohort of 98.9%, based on detected IgG cross-reactivity with the SARS-CoV-2 N protein in 1 of 88 controls.

#### Western blots

Crude insect cell lysates expressing either the N core domain or S ectodomain (see above) were separated on replica 10% SDS-PAGE gels and transferred to PVDF membranes according to standard protocols. Membranes were blocked with 1% milk powder and then probed with individual patient samples, diluted 1:50 in PBST containing 1% milk powder. Antigen-specific antibodies were detected by chemiluminescence using a rabbit anti-human IgG horseradish peroxidase conjugate; detected bands were compared to the expected molecular weight of the N and S proteins to confirm the specificity of detection. Antigen-specific IgG signals in individual patient samples were scored as strong, weak or absent by visual inspection of the western blots.

### Statistical analysis

Where relevant, descriptive statistics were utilized including mean and standard deviation and median and interquartile range. Analysis was performed on STATA 14 software (Statacorp, Texas, USA). Sensitivity and specificity were measured against the true positive results (defined by PCR) and negative results and expressed as percentage. A cumulative sensitivity and specificity were reported with 95% confidence interval (95% CI) and then the data were disaggregated by symptom score and by number of days post-PCR diagnosis (asymptomatic individuals) or post-symptom onset (symptomatic individuals). Reproducibility was measured utilizing 5 replicates with both high and low values run in the same run (intra-run precision) and on 5 separate days (inter-run precision) and reported as a % coefficient of variation (CV).

## Results

### Participant characteristics

The characteristics of the participants are listed in Table 1. In patients who provided their symptoms, 312/360 (86.7%) were symptomatic and 48/360 (13.3%) were asymptomatic. Symptoms were scored as mildly symptomatic (upper respiratory tract infections only), moderately symptomatic (lower respiratory tract symptoms, high fever or severe gastrointestinal symptoms) or severely symptomatic (requiring admission). Patients were also stratified according to the number of days post-PCR diagnosis (Table 1).

**Table 1:** Baseline characteristics of the study participants.

		Positive	Negative
Median Age in years (range), n = 368		41 (20–82)	54 (23–73)
Gender, (%)	Male	43%	43%
	Female	57%	57%
Ethnicity, (%)	African	33%	19%
	Caucasian	43.5%	76%
	Indian	13.7%	5%
	Mixed race	9.8%	0
Disease severity, n (%)	Asymptomatic	48(13.3%)	
	Mildly symptomatic	30(8.3%)	
	Moderately symptomatic	173(48.1%)	
	Severely symptomatic	109(30.3%)	
Days post-PCR diagnosis or post symptom onset, n = 375 (%)	0–7 days	76 (20.3%)	
	8–14 days	69 (18.4%)	
	15–21 days	47 (12.5%)	
	22–30 days	48 (12.8%)	
	31–40 days	35 (9.3%)	
	41–50 days	29 (7.7%)	
	>50 days	71 (19%)	

### Assay performance compared with RT-PCR results

All participants who had positive RT-PCR results were classified as true positive. The sensitivity of the IgG and IgA assays is summarized in Table 2. Performance was further disaggregated by days post-PCR diagnosis. The highest percentage positivity was reported at days 15–21 post-PCR diagnosis for IgG and days 31–40 for IgA; and the specificity was lower for IgA than IgG.

**Table 2:** Performance characteristics of EUROIMMUN Anti-SARS-CoV-2 IgA and IgG assays against RT-PCR.

		IgG	IgA
Sensitivity	Cumulative	64.1% (95% CI: 59.1–69.0%)	74.3% (95% CI: 69.6–78.6%)
	Day 0–7	34/75 (45.3%)	57/76 (75%)
	Day 8–14	38/69 (55%)	54/69 (78.2%)
	Day 15–21	38/47 (80.9%)	38/47 (80.9%)
	Day 22–30	24/48 (50%)	30/48 (62.5%)
	Day 31–40	27/35 (77.1%)	29/35 (82.9%)
	Day 41–50	21/29 (72.4%)	20/29 (68.9%)
	Day > 50	52/70 (74.2%)	48/71 (67.6%)
Specificity		95.2% (95% CI: 90.8–98.4%)	84.2% (95% CI: 77–89.2%)

The performance was further assessed according to the reported symptoms. The test positivity for all patients were then compared to PCR results. In individuals with moderate symptoms the positivity was highest (91.6%) at day 15–20 for IgG while in individuals with severe symptom the sensitivity was high (100%) at day31-50 for both IgG and IgA (Table 3).

**Table 3:** Assay performance of EUROIMMUN Anti-SARS-CoV-2 IgA and IgG Assay compared with PCR according to timepoint post-PCR diagnosis and disease severity.

	IgG (n = 355)							IgA (n = 355)						
Clinical presentation	Day 0–7 (n = 63)	Day 8–14 (n = 64)	Day 15–21 (n = 47)	Day 22–30 (n = 48)	Day 31–40 (n = 35)	Day 41–50 (n = 29)	Day > 50 (n = 71)	Day 0–7 (n = 63)	Day 8–14 (n = 64)	Day 15–21 (n = 46)	Day 22–30 (n = 48)	Day 31–40 (n = 35)	Day 41–50 (n = 29)	Day > 50 (n = 71)
Asymptomatic	28.5%	40%	50%	9%	66.7%	66.7%	80%	85.7%	80%	66.7%	33.3%	33.3%	33.3%	60%
Mild	NA^a^	33.3	NA^a^	50%	66.7%	NA^a^	66.7%	NA^a^	66.6%	NA^a^	66.6%	66.6%	NA^a^	66.7%
Moderate	27.2%	46.1%	**91.6%**	64%	76.9%	75%	72.7%	72.7%	69.2%	87.5%	80%	88.5%	81.3%	65.9%
Severe	61.9	66.7%	50%	66.7%	**100%**	**100%**	**91.7%**	78.6%	88.9%	92.3%	66.7%	**100%**	**100%**	**90%**

^a^NA- not available–No sample in the category for calculation

### Assay performance against in-house serology results

The sensitivity improved to over 80% when the assays was compared with in-house serology, however the specificity was reduced, with the cumulative IgA specificity at 73.8%. The highest positivity was reported at day>14 for IgG and day 0–14 for IgA (Table 4).

**Table 4:** Performance characteristics of EUROIMMUN Anti-SARS-CoV-2 IgA and IgG assays against in-house serology.

		IgG	IgA
Sensitivity	Cumulative	80% (95% CI: 71.5–86.9%)	87.8% (95% CI: 80.4–93.2%)
	Day 0–14	19/27 (70.4%)	25/27 (92.6%)
	Day>14	71/86 (82.6%)	75/86 (87.2%)
Specificity		86.9% (95% CI: 77.8–93.3%)	73.8% (95% CI: 63.1–82.8%)

The sensitivity was also evaluated by disease severity in comparison with in-house serology. The sensitivity of IgG and IgA was highest in day >14 post-symptom onset in both moderate and severely symptoms. The sensitivity of IgA (~ 90%) did not differ post days PCR/symptom onset in both moderate and severely symptoms (Table 5).

**Table 5:** EUROIMMUN Anti-SARS-CoV-2 IgA and IgG assays sensitivity stratified by days post-PCR diagnosis or symptom onset compared with In-house serology.

Clinical presentation	IgG (n = 111)		IgA (n = 111)	
	Day 0–14	Day >14	Day 0–14	Day >14
Moderate	8/11 (72.7%)	45/56 (80.3%)	10/11 (90.9%)	50/56 (89.3%)
Severe	8/10 (80%)	10/11 (90.9%)	9/10 (90%)	12/13 (92.3%)

### Precision analysis of the assays

For precision analysis, 5 samples were tested in duplicate on 5 consecutive days selected from both positive and negative samples. Precision was calculated for each assay utilising the comparative values and expressed as coefficient of variation (%CV). The % CV for IgA and IgG was 14,5% and 20.3%, respectively.

## Discussion

SARS-CoV-2 is the causative agent of COVID-19 disease. SARS-CoV-2 is one of the most highly pathogenic and transmissible coronavirus in humans, together with the severe acute respiratory syndrome coronavirus (SARS-CoV) and Middle East respiratory syndrome coronavirus (MERS-CoV) [46]. Accurate detection of SARS-CoV-2 infection is not only important for management of infected individuals but also to break the chain of transmission. In this study, we evaluated the diagnostic performance characteristics of the EUROIMMUN Anti-SARS-CoV-2 IgA and IgG assays.

The assay sensitivity was higher for IgA but low for IgG and improved for both assays in symptomatic individuals and at later timepoints post PCR diagnosis. The cumulative sensitivity for IgG is consistent other studies which report sensitivities ranging from 55.6–61.7% [47, 48]. Anti-SARS-CoV-2 IgG levels were low within 14 days after disease onset but subsequently increased to >80% between 15–21 days after onset of symptoms. This agrees with most other studies that reported low sensitivity prior to day 14 post-PCR [20, 48–54]. The sensitivity improved to over 80% when both Euroimmun IgA and IgG was compared with in house ELISA assays and more than 90% for severe patients at >day 14.

The lower sensitivity in individuals who are asymptomatic or mildly symptomatic is an important *caveat* to retrospective diagnosis and should be considered in public health responses to COVID-19. Although patients with asymptomatic disease may have lower viral loads, their potential infectious risk is unclear [26]. Other studies also reported significantly improved sensitivity in patients requiring admission [26, 55]. The study also reported a specificity lower than the manufacture’s package insert. Specificity lower than 90% for IgG [50, 51, 56] and IgA [47, 49, 51, 56] has been previously reported.

One of the factors that determines the reliability and accuracy of serological test is the choice of the targeted antigens. Currently, EUROIMMUN is the only automated serology platform authorized for use in South Africa which targets the S region and that includes IgA in this testing. IgA is regarded as a key mucosal antibody protecting from infection and neutralizing both bacterial and viral pathogens [57]. IgA is also secreted in body fluids such as saliva and blood. Most serology tests focus mainly on IgG and IgM, although IgA plays an important role in mucosal immunity. Several studies have reported high titre anti-SARS CoV-2 IgA in serum and with a higher sensitivity of IgA as compared to IgG [47, 49, 51, 58]. The IgA response appears as early as 2 weeks post symptom development [33, 59] and may be seen at similar timepoints to IgM of 5 (IQR, 3–6) days [28]. SARS-CoV-2 IgA antibodies are produced rapidly after infection and remain high in the plasma for longer after the onset of symptoms [23, 60, 61]. Studies have reported that antibody neutralization is associated with prolonged infection and RBD binding activity [62–64]. Additionally, the S protein has been considered as the ideal candidate antigen for inclusion in vaccines [65]. The vaccines currently authorized for emergency use by several regulatory agencies including U.S. Food and Drug Administration (FDA) target the spike protein [66, 67]. Although the FDA has removed EUROIMMUN IgA from the list of tests approved for emergency use because of its inferior specificity, specificity may be of less concern in an individual diagnostic assay than in broad seroprevalence studies [68].

There were a number of limitations in this study. Individuals self-reported their symptom onset dates, which might have led to error recall. Symptom data were also not available for all patients and this may explain the unexpected drop in sensitivity at day 21 of these 2 assays. At the time of the validation, there were no immune assay available targeting the spike protein for testing in South Africa and as a results we developed our own in house ELISA and Immunofluorescence assays to compare our findings. We feel, nevertheless, that this test shows good performance which should be considered in the context of a supplement diagnostic assay for SARS-CoV2.

This study recommends the use of EUROIMMUN Anti-SARS-CoV-2 IgA and IgG 1) as a complementary diagnostic tests after 14 days when PCR testing is most likely to become unreliable in individuals admitted with symptoms resembling SARS-CoV-2 disease, 2) in a specific subset of patients who are admitted with confirmed disease but who require antibody testing to confirm the presence of SARS-Co-2 multisystem inflammatory disorder or in patients admitted to intensive care for prognostication and 3) for seroprevalence surveys. In the context of the above recommendation, SARS-CoV-2 IgA and IgG will enable a more rapid and supplemental diagnostic approach to reduce the rapid spread of the SARS-CoV-2 globally [69].

## Supporting information

S1 Table EUROIMMUN IgG validations studies [70, 71]. (DOCX)

S2 Table EUROIMMUN IgA validation studies. (DOCX)

S1 Appendix Informed consent. (DOCX)
