# Supplementary material for: Performance of the EUROIMMUN Anti-SARS-CoV-2 ELISA Assay for detection of IgA and IgG antibodies in South Africa
Source: PLoS One. 2021 Jun 23;16(6):e0252317. doi: 10.1371/journal.pone.0252317 (PMC8221517; doi:10.1371/journal.pone.0252317)
Supplement: S1 Table — (DOCX) [file pone.0252317.s001.docx]

S1 Table: EUROIMMUN IgG validation studies

| Study | Number of samples tested | Sensitivity (cumulative and post-presentation where indicated) | Cumulative specificity |
| --- | --- | --- | --- |
| [29]  European | Patients:74  Controls:1248 | ≤10 days:43.7%  ≥10 days:94.4% | 99.6 % |
| [56]  United States of America | Patients:86  Controls:82 | 0-4 days: 97.7% (95%CI: 91.9–99.6%)  > 4 days: 100% (95%CI: 91.6–100%) | 67.1% (95%CI: 56.3–76.3%) |
| [54]  United States of America | Patients:56  Controls:224 | Days symptom onset  0-7 days: 0%  8-14 days: 27.5%  ≥15 days: 100%  Days post-PCR  0-7 days: 18.2%  ≥20 days: 91.3% | 98% (95%CI: 95.3% - 99.3%) |
| [49]  France | Patients:141  Controls:152 | Cumulative: 78% (95%CI: 70.9-84.3%)  0-7 days: 28.1% (95%CI: 42.3-74.5%)  8-14 days: 72.4% (95%CI: 54.3-85.3%)  ≥15 days: 100% (95%CI: 95.5-100%) | 96.7 (95%CI: 92.4-98.6%) |
| [70]  Austria | Patients:73  Controls:100 | 1-5days: 3.3%  6-10 days :40%  ≥11 days:100% | 98% |
| [48]  Belgium | Patients:167  Controls:103 | Cumulative: 55.6%(95%CI: 47.6-63.2%)  0-6 days: 21.6% (95%CI: 11.1-37.4%)  7-13 days :55.6% (95%CI: 47.6-63.2%)  14-25 days :89.5% (95%CI: 75.3-96.4%) | 96.1%(95 % CI: 90.1-98.8%) |
| [53]  Italy | Patients:171 | ≤5 days: 0%  >5–10 days:15.4%  >10–21 days: 100% | NA |
| [39]  Germany | Patients:45  Controls:37 | 71.1% | 100% |
| [71]  Germany | Patients:33  Controls:18 | 5-9 days: 58.8%  10-18 days: 93.8 % | 95.7% |
| [47]  Belgium | Patients:172  Controls:82 | Cumulative: 61.7% (95%CI: 53.1-69.7%) | 98.6%(95 % CI: 92.5-99.8%) |
| [51]  France | Patients:38  Controls:20 | Cumulative:85% (95%CI: 70.4 - 99.6%) | 93.3% (95 % CI: 80.7 - 100%) |
| [58]  United States of America | Patients:100  Controls:300 | Cumulative:82.8% (95%CI: 75.4-90.2%) | 99.7% (95 % CI: 99.1-100%) |
| [50]  Australia | Patients:91  Controls:92 | <14 days: 56.2%(95%CI: 47.5–64.7%)  >14 days: 92.3%(95%CI: 83.0–97.5%) | <14 days: 97.8% (95 % CI: 92.4–99.7%)  >14 days: 97.8%(95 % CI: 92.4–99.7%) |
| [20]  United States of America | Patients:103  Controls:156 | Days symptom onset  <3days :0.0% (95%CI: 0–26.47%)  3–7 days: 25%(95%CI: 8.66–49.10%)  8–13 days:56.5% (95%CI:34.49–76.81%)  ≥14 days 85.4% (95%CI: 72.24–93.93%)  Days post-PCR  <3 days:38.1% (95%CI: 23.57–54.36%)  3–7 days: 63.6% (95%CI: 40.66–82.80%)  8–13 days:69.6% (95%CI: 47.08–86.79%)  ≥14 days:75% (95%CI:47.62–92.73%) | 94.8% (95% CI:89.96–97.72%) |

NA-Not applicable
