# Supplementary material for: Performance of the EUROIMMUN Anti-SARS-CoV-2 ELISA Assay for detection of IgA and IgG antibodies in South Africa
Source: PLoS One. 2021 Jun 23;16(6):e0252317. doi: 10.1371/journal.pone.0252317 (PMC8221517; doi:10.1371/journal.pone.0252317)
Supplement: S2 Table — (DOCX) [file pone.0252317.s002.docx]

Table S2: EUROIMMUN IgA validation studies

| Study | Number of samples tested | Sensitivity | Cumulative specificity |
| --- | --- | --- | --- |
| [30]  European | Patients:152  Controls:1332 | ≤10 days:60.2%  ≥10 days: 98.6% | 92% |
| [56]  United States of America | Patients:86  Controls:92 | 0-4 days: 82.9% (95%CI: 73.4–89.5%)  >4 days: 90.5% (95%CI: 77.9–96.2%) | 88.4% (95%CI: 79.9–93.6%) |
| [49]  France | Patients:141  Controls:142 | Cumulative:86.7% (95%CI: 80.2-91.3%)  0-7 days: 59.4% (95%CI: 15.6-45.4%)  8-14 days: 79.3% (95%CI: 61.6-90.2%)  ≥15 days: 100% (95%CI: 95.5-100%) | 82.7% (95%CI: 75.8-87.9%) |
| [70]  Austria | Patients:73  Controls:100 | 1-5days: 30%  6-10 days :84%  ≥11days:100% | 98% |
| [53]  Italy | Patients:171 | ≤5 days: 3.3%  >5–10 days: 30.8%  >10–21 days:100% | NA |
| [47]  Belgium | Patients:172  Controls:82 | Cumulative: 83.6% (95%CI: 76.2-89%) | 86.1% (95%CI: 76.3-92.3%) |
| [51]  France | Patients:38  Controls:20 | Cumulative: 93.3% (95%CI: 80.7 - 100%) | 80.0% (95%CI: 63.7 - 96.3%) |
| [58]  United States of America | Patients:100  Controls:300 | Cumulative: 95.0% (95%CI: 90.7-99.3%) | 93.7% (95%CI: 90.9-96.5%) |
| [50]  Australia | Patients:91  Controls:92 | <14 days: 65.7%(95%CI: 57.1–73.6%)  >14 days: 89.2% (95CI%: 79.1–95.6%) | <14 days: 73.9% (95%CI: 63.7–82.5%)  >14 days: 73.9%(95%CI: 63.7–82.5%) |

NA-Not applicable
