# Supplementary material for: Performance of the EUROIMMUN Anti-SARS-CoV-2 ELISA Assay for detection of IgA and IgG antibodies in South Africa
Source: PLoS One. 2021 Jun 23;16(6):e0252317. doi: 10.1371/journal.pone.0252317 (PMC8221517; doi:10.1371/journal.pone.0252317)
Supplement: S1 Appendix — (DOCX) [file pone.0252317.s003.docx]

Questionnaire for patients

|  |  |  |  |  |
| --- | --- | --- | --- | --- |
| **Demographic data** | | | | |
| Age | 18-25 years | 25-40 yrs | 40-60 yrs | >60 yrs |
| Gender | Female | Male | Other |  |
| Ethnicity | African | Coloured | Caucasian | Indian |
| **Comorbid conditions** | | | |  |
| Diabetes | Type 1 | Type 2 |  |  |
| Hypertension | Yes | No |  |  |
| Cancer | Haematological  Specify: | Solid organ  Specify |  |  |
| Chronic lung diseases | Yes | No | Specify: |  |
| Asthma | Yes | No |  |  |
| Rheumatological conditions | Yes | No | Specify |  |
| **Medications** | | | | |
| Steroids e,g, prednisone | Yes | No | Form of administration  IV/Topical/Oral: | Dose: |
| Anti-inflammatory agents e.g. aspirin, ibuprofen | Yes | No | Form of administration  IV/Topical/Oral: |  |
| Antiviral agents e.g. Tamiflu | Yes | No | Form of administration  IV/Topical/Oral: |  |
| Antihypertensives (for high blood pressure e.g. Ridaq, pharmapress) | Yes | No | Form of administration  IV/Topical/Oral: |  |
| Antibiotics e.g. penicillin | Yes | No | Form of administration  IV/Topical/Oral: |  |
| Biological agents e.g infliximab for rheumatoid arthritis | Yes | No | Form of administration  IV/Topical/Oral: |  |
| Chemotherapy for cancer | Yes | No | Form of administration  IV/Topical/Oral: |  |
| Hormonal therapy including use of the oral contraceptive pill or injection | Yes | No | Form of administration  IV/Topical/Oral:  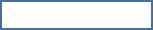 |  |
| **Symptom** | **Duration** | **Severity** |  |  |
| Cough |  |  |  |  |
| Runny nose |  |  |  |  |
| Sore throat |  |  |  |  |
| Fever |  |  |  |  |
| Headache |  |  |  |  |
| Generalised body pain |  |  |  |  |
| Diarrhoea |  | Number of loose stools: |  |  |
| Vomiting/nausea |  |  |  |  |
| Admitted | Yes | No | General ward | ICU |
| **Travel history (only within last 30 days prior to seeking clinical care)** | | | |  |
| None |  |  |  |  |
| Within South Africa |  |  |  |  |
| In Africa |  |  |  |  |
| Europe |  |  |  |  |
| US |  |  |  |  |
| China |  |  |  |  |
| Asia (excluding China) |  |  |  |  |
| Other |  |  |  |  |

**Information document for patients**

Good Day,

My name is Elizabeth Mayne and I am working with some of the clinicians and virologists who are assisting to diagnose, treat and control the current outbreak of the SARS-CoV2 (COVID-19) virus. This is a virus which is highly contagious and which can spread rapidly through droplets. You have been identified as a patient who is at high risk for having contracted the virus or you have tested positive with the virus. We would like to request that you assist us to develop rapid diagnostic techniques to diagnose patients who are infected. This will help us identify which individuals should be quarantined and hopefully assist in reducing transmission in South Africa.

**How you can help?**

We would like to request that you donate the following samples to us at the time you are seen by the doctor treating you and if possible 1 and 2 weeks later:

1. At least 8 ml (1 tube/collection jar) of saliva (about 2 teaspoons)
2. At least 2 swabs of inside your mouth and 1 of the inside of your nose
3. 2-6 full red or yellow topped (serum) tubes (approximately 2-6 teaspoons of blood) depending on your clinical symptoms
4. 2 full purple topped (EDTA) tubes (approximately 2 teaspoons of blood)

We will also ask you to answer a few questions regarding your travel history, your clinical symptoms, your contact history, any medical conditions which you may have and your current medications.

**What will we do with the samples you provide?**

The samples will be used to evaluate the performance of rapid tests which use either identification of the virus (on a swab or in your saliva) or antibodies that your body makes against the virus. In addition, we are evaluating a laboratory test which will assist in identifying the antibody response in patients with the virus. Any residual blood or other material will be stored in a biorepository in Johannesburg, so that we have known positive samples that we can use to assess the quality of our testing and for identification of additional tests that may assist in controlling the disease. The testing that will be undertaken will include testing for blood chemicals (called cytokines) which may be associated with severe disease, additional testing for anti-COVID antibodies, testing for COVID antigen by several different rapid tests and characterisation of the white blood cells in your blood which could assist in fighting the infection. Your samples will be stored under strictly controlled conditions and all of the data linking the data with your identity will be protected. It may become necessary to transfer some of the material to laboratories around the country but this will be under strict control. You will be able to withdraw your material from storage if you are concerned about this aspect.

**What are the risks associated with participating in the study?**

The study will take place under strict safety conditions to protect both you and the doctors who will be taking the samples. Venous blood draw may be associated with transient pain. Your name and clinical details that you provide will be anonymised and will only be known to the doctors who are responsible for your clinical care and the laboratory which is receiving your samples and making the diagnosis. Any other data will be made anonymous.

**What is the benefit to you of participating in this study?**

There is no direct benefit to you. We will not reveal the results of your testing to you as the test must be properly evaluated before it can be used to make a diagnosis. The benefit will be to identify rapid tests that may help us control the viral spread and also to see what the immune response of South Africans is to the virus.

**Will this affect my care?**

This will have no affect at all on your clinical care. Participation is voluntary and you can withdraw at any time without affecting your clinical care or diagnosis. Please be aware that COVID-19 is a notifiable disease and that all correct clinical protocols must be followed (this includes contact tracing). Please note in addition, that the request to participate in this study does not mean that you are infected or are likely to become infected.

**Contact details for any queries or concerns: This study has been approved by the Human Research Ethics Committee (Medical) of the University of the Witwatersrand. If you have any queries or concerns please contact:**

Elizabeth Mayne

Office: 011 489 8862

Mobile: 082 337 6349

Email: elizabeth.mayne@nhls.ac.za

Human Research Ethics Committee:

Dr Clem Penny

E mail: Clement.Penny@wits.ac.za

Informed Consent:

I, (patient name), acknowledge that I have read and understand the informed consent document (or that I have been explained the contents in a language that I understand. I hereby agree to the donation of samples for the purposes specified and for completion of the clinical questionnaire. I also acknowledge that I understand that this will have no impact on my clinical care. I understand that I have the right to withdraw my consent at any time.

| Patient name |  |
| --- | --- |
| Patient signature |  |
| Witness signature |  |
| Date |  |

Storage consent for blood and saliva samples

I, (patient name), acknowledge that I have read and understand the informed consent document (or that I have been explained the contents in a language that I understand. I hereby agree to the storage of my blood and saliva samples at Contract Laboratory Services, an approved biobank in Johannesburg; for the purposes specified, that is for evaluation of new testing for COVID-19, for evaluation of immunological responses and to assess the quality of tests which are performed by diagnostic laboratories. For these purposes, it may be necessary to transfer samples to testing laboratories but this will be under strict control. At all times, my personal information will be protected and kept confidential. I also acknowledge that I understand that my consent will have no impact on my clinical care. I understand that I have the right to withdraw my consent at any time.

| Patient name |  |
| --- | --- |
| Patient signature |  |
| Witness signature |  |
| Date |  |
